# Unveiling the consequences of early human saliva contamination on membranes for guided bone regeneration

**DOI:** 10.1111/jre.13266

**Published:** 2024-04-22

**Authors:** Marcel F. Kunrath, Paula Milena Giraldo‐Osorno, Karina Mendes, Ana T. P. C. Gomes, Nuno Rosa, Marlene Barros, Christer Dahlin

**Affiliations:** ^1^ Department of Biomaterials, Institute of Clinical Sciences, Sahlgrenska Academy University of Gothenburg Göteborg Sweden; ^2^ Center for Interdisciplinary Research in Health (CIIS), Faculty of Dental Medicine (FMD) Universidade Católica Portuguesa Viseu Portugal; ^3^ Dentistry Department, School of Health and Life Sciences Pontifical Catholic University of Rio Grande do Sul (PUCRS) Porto Alegre Brazil

**Keywords:** biocompatibility, dental materials, guided regeneration, oral regeneration, periodontology, saliva

## Abstract

**Aims:**

GBR membranes have various surface properties designed to elicit positive responses in regenerative clinical procedures; dental clinicians attempt to employ techniques to prevent the direct interaction of contaminated oral fluids with these biomaterials. However, saliva is uninterruptedly exhibited in oral surgical procedures applying GBR membranes, suggesting a persistent interaction with biomaterials and the surrounding oral tissues. This fundamental study aimed to investigate potential alterations in the physical, chemical, and key biological properties of membranes for guided bone regeneration (GBR) caused by isolated early interaction with human saliva.

**Methods:**

A reproducible step‐by‐step protocol for collecting and interacting human saliva with membranes was developed. Subsequently, membranes were evaluated for their physicochemical properties, protein quantification, DNA, and 16S rRNA levels viability of two different cell lines at 1 and 7 days, and ALP activity. Non‐interacted membranes and pure saliva of donors were applied as controls.

**Results:**

Qualitative morphological alterations were noticed; DNA extraction and 16S quantification revealed significantly higher values. Furthermore, the viability of HGF‐1 and MC3T3‐E1 cells was significantly (*p* < .05) reduced following saliva interaction with biodegradable membranes. Saliva contamination did not prejudice PTFE membranes significantly in any biological assay.

**Conclusions:**

These outcomes demonstrated a susceptible response of biodegradable membranes to isolated early human saliva interaction, suggesting impairment of structural morphology, reduced viability to HGF‐1 and MC3T3‐E1, and higher absorption/adherence of DNA/16S rRNA. As a result, clinical oral procedures may need corresponding refinements.

## 1 INTRODUCTION

Membranes for guided bone regeneration (GBR) have been used in oral regenerative procedures on a large scale during the last several decades.^1^, ^2^, ^3^ Clinical success using several membrane types has been achieved in challenging cases requiring bone augmentation, sinus lift, coverage of bone defects, and coverage of implant surfaces.^1^, ^3^, ^4^, ^5^, ^6^ Current GBR membranes have highly bioactive surface properties that promote bone regeneration and prevent soft tissue invasion into the bone–tissue complex. However, the success of such treatments depends on a careful handling procedure to avoid the interaction of any contaminants within the system.^7^, ^8^ Changes in the physicochemical and biological properties of GBR membranes caused by early saliva contamination are not clearly described in the current literature.

Saliva is an oral fluid composed of water, minerals, and proteins present in almost all oral procedures, except for patients with underlying systemic diseases preventing saliva production. However, once excreted from the salivary ducts, saliva interacts with bacteria, blood, and cells, changing its composition.^9^, ^10^, ^11^ Application of GBR membranes without any contact with oral fluid during the surgical procedure remains challenging,^12^ and the likelihood of unwanted contact of membranes and saliva may be increased due to: (1) inattention during the surgical handling, (2) insufficient cleaning of the surgical site, (3) presence of hypersalivation, (4) working with disabled patients, (5) performing periodontal surgeries, and others.^12^, ^13^ In addition, several surgical procedures might expose GBR membranes to saliva after late gingival dehiscence/failure, resulting in unplanned saliva interaction and contamination.^14^, ^15^

These initial, continuous, or late interactions with saliva may generate internal and/or external physicochemical changes on the membranes, thus interfering with the early biological responses and capacity of bone regeneration. Recently, a fundamental study investigated the effects of artificial saliva interaction with three different biodegradable membranes, demonstrating negative outcomes for wettability, tensile strength, and degradation rates compared with non‐interacted membranes.^16^ An in vitro study demonstrated that the interaction of saliva with murine bone marrow cells suppressed osteoclastogenesis.^17^ Another study using MC3T3‐E1 cell line showed impairment of cell performance after saliva exposure owing to the presence of enzymes in the oral fluid.^18^ Furthermore, saliva has been shown to carry molecules, including endotoxins, which can initiate pro‐inflammatory activity in macrophages.^19^ Taken together, the above‐mentioned findings show that the isolated saliva interaction with primary cells exhibited in sites of GBR procedures may have consequences on the biological response and planned clinical outcomes.

Identifying which physicochemical and fundamental biological properties of GBR membranes are affected by the early saliva interaction is of paramount importance to clinicians and the dental industry in order to improve manipulation, intervention, and/or re‐treatment of oral defects. Our null hypothesis is that isolated early human saliva interaction does not cause any property alteration in GBR membranes. Thus, this in vitro study using human saliva aimed to demonstrate the tentative changes in key physical, chemical, and biological properties caused by isolated early human saliva interaction with membranes for GBR, evaluating important membrane properties tailored for successful clinical treatment in patients.

## 2 METHODS

### 2.1 GBR membranes

To investigate the physicochemical and biological alterations on GBR membranes caused by interaction with saliva, three different commercially available membranes presenting resorbable and non‐resorbable properties were selected. A biodegradable collagen membrane (Cytoplast™ RTM Collagen, Osteogenics Biomedical Inc., Texas, USA); a non‐biodegradable membrane manufactured with e‐PTFE (NeoGen™, Neoss Ltd, UK); and a non‐biodegradable membrane manufactured with d‐PTFE (Cytoplast™ TXT‐200, Osteogenics Biomedical Inc., Texas, USA). The different membrane types were punched (⌀ 10 mm) under a laminar flow hood using sterilized biopsy punches (Acu Punch, Acuderm Inc., Florida, USA). Membranes were stored in sterilized packages for immediate use in subsequent experiments.

### 2.2 Saliva collection and interaction

**FIGURE 1:**
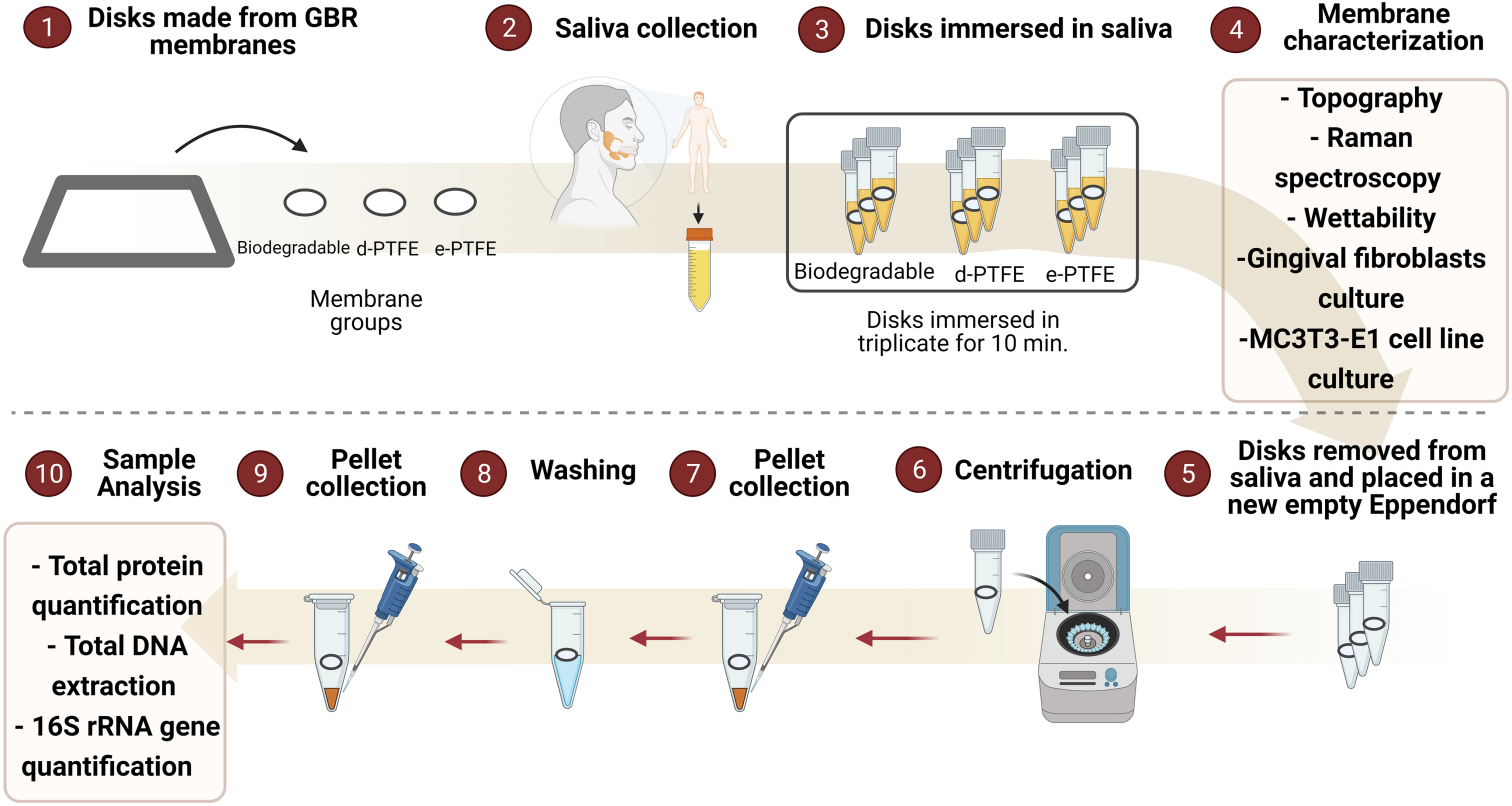
(1–10) Scheme demonstrating the experimental methods applied in this study; (5–10) Methodology to detach saliva content from membranes for GBR after saliva interaction.

Unstimulated whole saliva was collected from three donors with excellent oral conditions, considered healthy donors (35 years old, male; 25 years old, female; 23 years old, female). The definition and eligibility criteria of “healthy donors” was determined by previous clinical evaluation checking the non‐presence of caries, non‐presence of periodontal pockets, and/or non‐presence of gingivitis; and the non‐exhibition of metabolic diseases and/or use of medications, checked by previous anamnesis. This study has the approval of the Ethics Committee of Universidade Católica Portuguesa—Viseu, Portugal (protocol no. CES‐UCP/113) and followed the standards of Declaration of Helsinki. In brief, whole saliva was collected in 50 mL sterile centrifuge tubes and immediately used in the subsequent experiments preventing the loss of biological properties. To contaminate, the GBR membranes with saliva same procedure was used throughout this study. Disk‐shaped membranes were placed in sterile 1.5 mL centrifuge tubes and completely immersed in 300 μL of saliva. Centrifuge tubes with submerged membranes were shaken in a thermal shaker (Thermal Shake *lite*, Avantor™, Portugal) using a speed of 300 min^−1^ at 37°C for 10 min (Figure 1). Pure saliva collected from the patients was applied as a control for the extraction assays. This study followed the modified CONSORT checklist suggested for preclinical in vitro studies on dental materials.^20^

### 2.3 Physiochemical characterization

For topographical characterization, membranes were investigated using light optical microscopy (Nikon Eclipse E600, Nikon®, Japan) and scanning electron microscopy (SEM, Inspect F50, Prague, Czech Republic). To determine roughness characteristics, Ra (arithmetic roughness average) and Rq (root mean square roughness) parameters were investigated using an atomic force microscope (AFM, Dimension Icon, Bruker, Billerica, MA, USA). A confocal Raman microscope (Renishaw inVia Qontor, Renishaw plc. Wotton under Edge, UK) was used for micro‐Raman spectroscopy of different membrane types to identify possible saliva‐induced alterations. Measurements were made using a 633 nm laser focused through a 50×/0.50 NA objective, and the Raman scattered light was collected using a Peltier‐cooled charge‐coupled device deep depletion near‐infrared enhanced detector behind an 1800 grooves/mm grating. For all samples, six different positions were analyzed with 0.5 or 0.1 s integration time and five accumulations in biodegradable or PTFE membranes, respectively. Raw data were processed using Renishaw Wire 5.4 software by performing background subtraction and cosmic ray removal. Collected spectra were averaged using the same software. Physics characteristics such as membrane thickness and density were evaluated according to previous study^21^ before and after saliva exposure.

The wettability of membranes was measured using a Goniometer—Contact Angle Measure (Phoenix 300, SEO, Kosekdong, Korea) and software (Surfaceware8, version 10.11, Korea). In short, 10 μL of deionized water was dropped onto the disk‐shaped membranes, before and after saliva contamination (*n* = 3 per membrane type/per saliva donor) and the contact angles were evaluated.

### 2.4 Saliva proteins adherence to membranes after saliva contamination (Protein Quantification by Bicinchoninic Acid Assay)

GBR membrane adhered/absorbed salivary proteins were quantified according to the protocol described in Section 2.2. The membrane disks (*n* = 3 per group/per saliva donor) were transferred into a clean 1.5 mL centrifuge tube and centrifuged at 16 000*g* for 10 min at RT. Following the centrifugation, membranes were discarded. The recovered volume was treated with 50 μL of 1% (w/v) sodium dodecyl sulfate (SDS, Sigma‐Aldrich, Germany) in phosphate‐buffered saline solution (PBS, NZYtech, Portugal) at pH 7.0, and vortexed for 30 s. The concentration of proteins in each membrane was determined in triplicate by a bicinchoninic acid assay using the Pierce™ BCA Protein Assay Kit (Thermo Fisher Scientific, USA) according to the manufacturer's protocol. Protein quantification was determined using pure saliva samples without membranes as a control.

### 2.5 Total DNA quantification and total bacterial load adhered/absorbed to saliva‐contaminated membranes

Total bacterial load in saliva‐contaminated membranes was determined by quantifying the 16S rRNA gene using quantitative real‐time PCR (qRT‐PCR). Saliva‐contaminated membrane disks (Section 2.2) were transferred into clean 1.5 mL centrifuge tubes and centrifuged at 16 000 *g* for 10 min at RT. After centrifugation, membranes were discarded and DNA was extracted from the recovered volume using the QuickExtract™ DNA Extraction Solution (QE0905T, Lucigen, USA). Briefly, to isolate DNA, 50 μL of QuickExtract solution was added to the recovered saliva, followed by quick vortex (5 s) and incubation for 6 min at 65°C and for 2 min at 98°C. Isolated DNA was quantified using the μDrop Plate (Thermo Fisher Scientific). Total bacterial load by qRT‐PCR was determined using universal primers targeting bacterial 16S rRNA gene (926F: 5′ AAACTCAAAKGAATTGACGG 3′; 1062R: 5′ CTCACRRCACGAGCTGAC 3′).^22^ Each DNA sample was analyzed in duplicate. The total volume of an individual PCR reaction was 10 μL. Each reaction consisted of 5 μL of NZYSpeedy qPCR Green Master Mix (2×) (MB224, NZYtech, Portugal), 0.4 μM of forward, 0.4 μM of reverse primer, and 1 ng of sample DNA. Amplification and detection were performed in the CFX96 Touch Real‐Time PCR Detection System (Bio‐Rad, Portugal) using the following thermocycling conditions: 95°C for 3 min (1 cycle), 95°C for 5 s, and 61.5°C for 20 s (40 cycles), and infinite hold at 4°C. To perform absolute quantification of the total bacterial load, a standard curve was generated using plasmid DNA of *Staphylococcus capitis* containing a known concentration of 16S rRNA gene. Duplicate, 10‐fold dilutions of plasmid DNA corresponding to seven non‐zero standard concentrations were used, from 2.77 × 10^10^ to 2.77 × 10^4^ copies of DNA per reaction. The copy numbers of the 16S rRNA for each sample were calculated from the standard curve. Pure saliva samples from the three patients were used as controls.

### 2.6 Human gingival fibroblasts viability and cytotoxicity before and after membrane saliva contamination

The human gingival fibroblast cell line HGF‐1 (ATCC CRL‐2014, Manassas, USA) was grown in Dulbecco's modified Eagle's medium supplemented with 10% FBS and 1% penicillin/streptomycin solution (Gibco Life Technologies, Carlsbad, California, USA) in a 37°C humidified incubator with 5% CO_2_. HGF‐1 were seeded at a density of 40 000 cells/mL on the surface of the membrane disks (*n* = 3 per membrane group/per saliva donor) juxtaposed in Nunc non‐adherent 48‐well plates (Thermo Fisher Scientific, Roskilde, Denmark) with 300 μL media. The number of adhered cells was assessed after 24 and 168 h. The membrane disks were gently transferred to an empty well and washed twice with PBS to remove non‐adherent/nonviable cells before adding NucleoCounter® lysis and stabilization buffer in equal volume. Lysed cells were loaded into a Nucleocassette™ containing immobilized propidium iodide, which stains cell nuclei of the lysed sample, and the results were quantified using a NucleoCounter® NC‐200 (ChemoMetec A/S, Allerod, Denmark). Cell toxicity was further analyzed by quantifying the lactate dehydrogenase (LDH) content in the media using an LDH activity kit according to the manufacturer's instructions (Sigma‐Aldrich, Munich, Germany).

### 2.7 Preosteoblasts cell line viability and cytotoxicity before and after membrane saliva contamination

For the bone‐related cell investigation, a mouse preosteoblast cell line MC3T3‐E1 was applied. Preosteoblasts were maintained in normal culture medium a‐MEM, supplemented with 10% fetal calf serum, 4500 mg glucose, 0.1% gentamycin, and 0.1% ascorbic acid under standard culture conditions (37°C, 5% CO_2_ in a humidified atmosphere). The medium was refreshed every 48 h. After reaching confluence, cells were detached from culture vessels by incubating with pronase for 3–5 min. MC3T3‐E1 cells were seeded at a density of 40 000 cells/mL on the surface of the membrane disks (*n* = 3 per membrane group/per saliva donor) juxtaposed in non‐adherent 48‐well plates (Thermo Fisher Scientific, Massachusetts, USA) with 300 μL media.

The number of adhered cells was assessed after 24 and 168 h. The membrane disks were gently transferred to an empty well and washed twice with PBS to remove non‐adherent/nonviable cells before adding NucleoCounter® lysis and stabilization buffer in equal volume. Lysed cells were loaded into a Nucleocassette™ containing immobilized propidium iodide, which stains the cell nuclei of the lysed sample. The results were investigated as described in the previous chapter 2.6.

To investigate alkaline phosphatase (ALP) activity, preosteoblast cells were seeded into 48‐well plates (Thermo Fisher Scientific, Massachusetts, USA), on the different membrane groups (contaminated with saliva and non‐contaminated), and incubated at 37°C with 5% CO_2_. The intra‐cellular ALP was measured at 1 and 7 days. Afterwards, centrifugation at 11180 *g* force for 5 min was performed and the supernatant was collected to detect ALP activity. The final measurements were normalized using the total protein quantification and calculated using a BCA (bicinchoninic acid) Protein Assay Kit (Pierce, Bonn, Germany) according to the manufacturer's instructions. Final absorbance was investigated using a spectrophotometric microplate reader at 490 nm.

### 2.8 Statistical analysis

Data are presented as mean ± standard deviation (SD). Experiments were performed in triplicates for all analyses, using a triplicate of saliva donors generating statistical significance. For continuous variables such as wettability, roughness, thickness, membrane density, protein quantification, genetic load, and HGF‐1/MC3T3‐E1 assays, comparisons between non‐contaminated, and saliva‐contaminated groups were made using Student's *t*‐test. To compare outcome variables within the three groups of membranes, when necessary, one‐way ANOVA was applied to analyze the final results (biological assays). Statistical analysis and graph design were performed using GraphPad Prism 9.0 (California, San Diego, USA), and the level of significance applied was 0.05 (*p* < .05).

## 3 RESULTS

The null hypothesis of this study was rejected. Early saliva interaction with three distinct membrane types has altered significantly key membrane properties at different levels as detailed below.

### 3.1 Physical–chemical characterization

#### 3.1.1 Morphology and chemical composition

**FIGURE 2:**
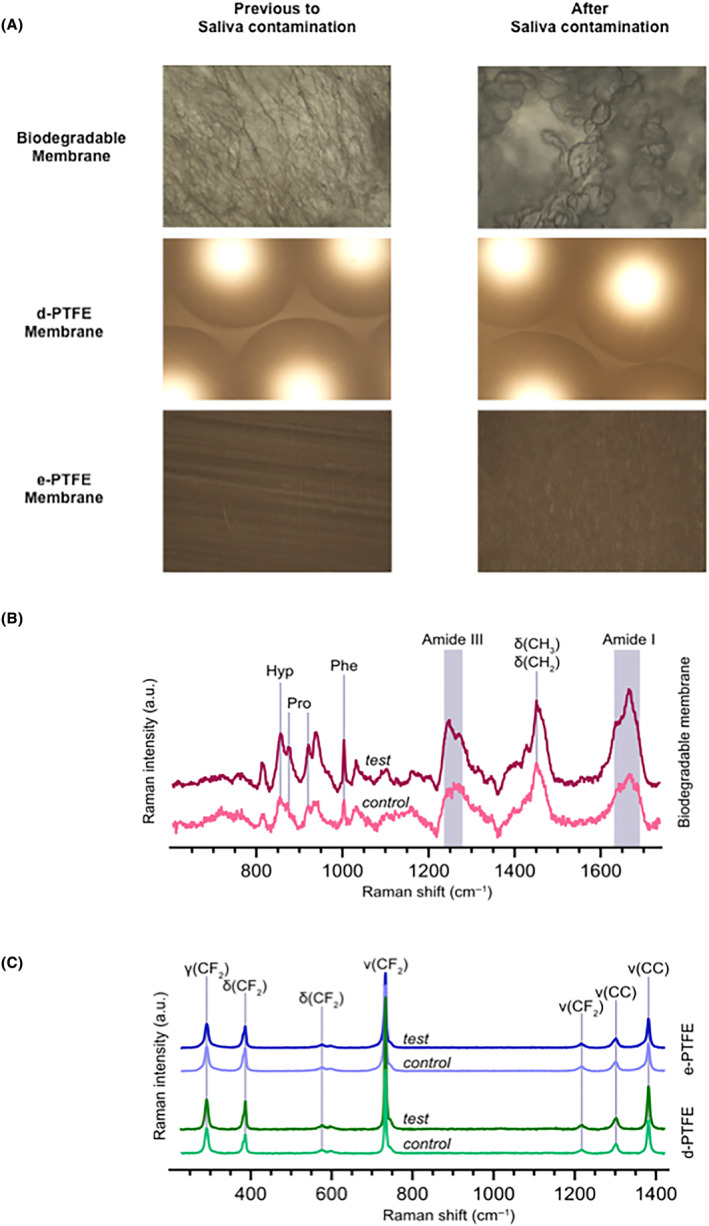
Light optical microscopy showing qualitative morphological characteristics of the different membrane types tested in this study (10× magnification) (A). Raman spectra comparison of saliva‐contaminated and as‐manufactured collagen biodegradable membranes. Characteristic bands of collagen are present in both membrane samples: Hyp—hydroxyproline, Pro—proline, and Phe—phenylalanine (B). Raman spectra comparison of saliva‐contaminated and as‐manufactured d‐PTFE (dense) and e‐PTFE (expanded) membranes. Characteristic bands of PTFE are present in all instances (C).

Qualitative morphological characteristics were investigated using optical microscopy and scanning electron microscopy. Unsurprisingly, the fibrous structure of the biodegradable membrane changed with the addition of saliva to reflect a swollen fiber morphology. No significant morphological changes were noticed in the non‐biodegradable membranes using optical microscopy (Figure 2A). Similar qualitative characterization was demonstrated between the different saliva donors, resulting in resembling data.

Comparable Raman spectra were observed in both saliva‐contaminated (test) and as‐manufactured biodegradable collagen membranes (control) (Figure 2B). Collagen characteristic bands can be assigned as hydroxyproline at 872 cm^−1^, proline at 854 and 920 cm^−1^, phenylalanine at 1003 cm^−1^, amide III in 1243–1270 cm^−1^ region, δ(CH2) and δ(CH3) at 1447 cm^−1^, and amide I in 1636–1668 cm^−1^ region. No significant distinction in chemical composition was identified between saliva‐contaminated and control samples. Raman analyses of PTFE membranes (d‐PTFE and e‐PTFE) that underwent identical saliva contaminations showed no influence of saliva on the chemical composition of membranes. Highly stable PTFE membranes showed distinguishable *fingerprints* with peaks representing deformation of CF2 at 292 cm^−1^, bending at 385 and 574 cm^−1^, and stretching at 731 and 1216 cm^−1^. As well as, stretching of CC at 1300 and 1377 cm^−1^ (Figure 2C).

**FIGURE 3:**
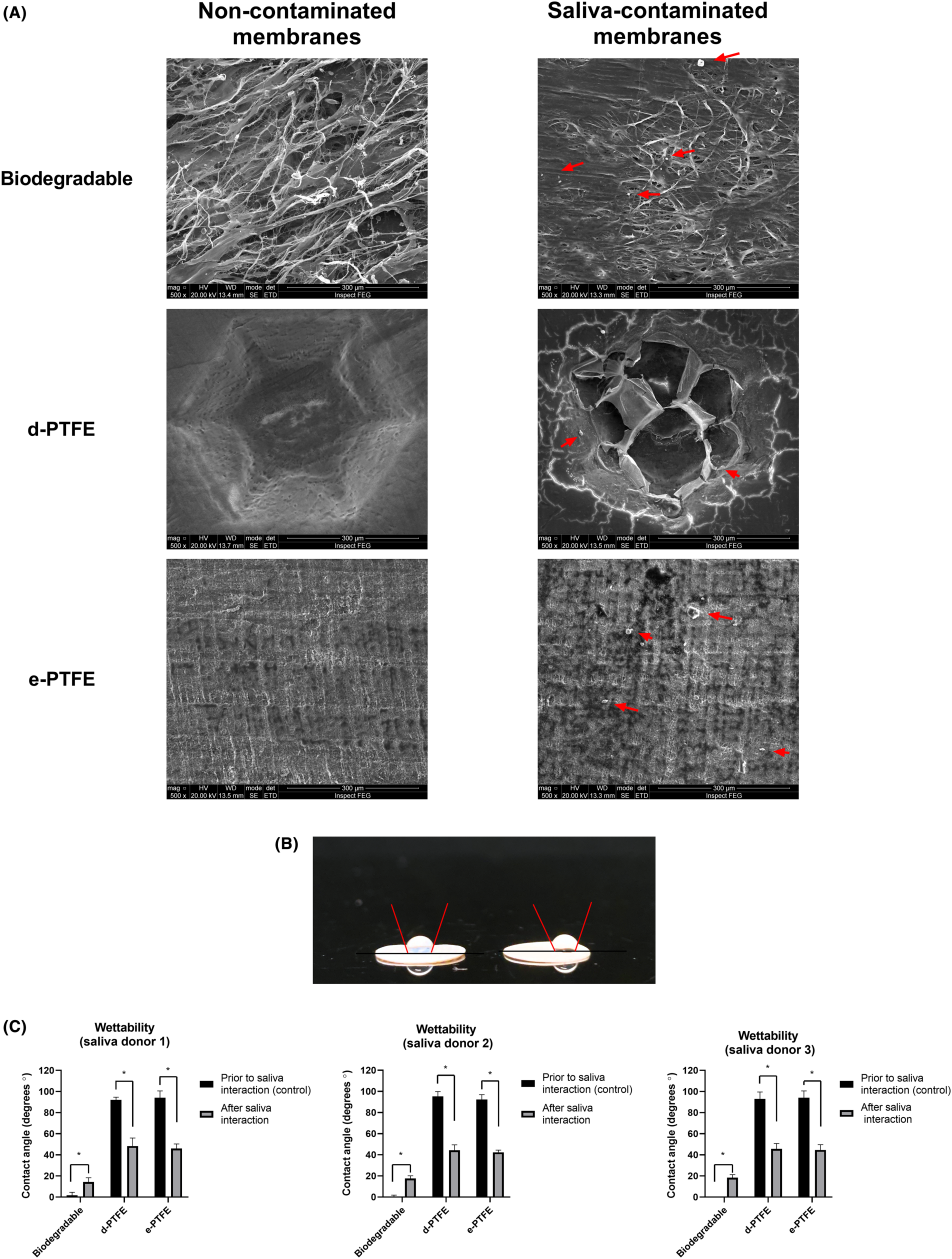
Scanning electron microscopies showing the three different membrane groups before and after saliva interaction. Apparent contamination and impurities derived from saliva were noticed on the three membrane groups after saliva interaction (red arrows) (A) (SEM magnification applied 500×—scale bar = 300 μm). Example of the wettability test performed on d‐PTFE and e‐PTFE membranes (B). Evaluation of wettability measurements before and after saliva interaction with different saliva donors (C). * symbolizes statistical significance (*p* < .05).

Scanning electron microscopies revealed evident impurities for the three membrane groups after saliva interaction. Saliva‐contaminated biodegradable membranes showed qualitative differences compared with their controls. Collagen fibers changed orientation and density after saliva exposure, associated with impurities detected in internal and external areas. Meanwhile, for PTFE‐membrane groups were only possible to notice external saliva contamination (Figure 3A).

#### 3.1.2 Wettability and membrane features

Firstly, biodegradable and non‐biodegradable membranes demonstrated an opposite wettability profile (hydrophilic and hydrophobic, respectively) when tested in their direct commercial status. The biodegradable collagen membrane showed hydrophilic conditions (~0° ± 2°) before the interaction with saliva and a slight decrease in hydrophilic characteristics after the exposure (~12° ± 3°). Meanwhile, both, non‐biodegradable membranes (d‐PTFE and e‐PTFE) demonstrated hydrophobic conditions (~95° ± 4°) before the interaction with the saliva and a significant reduction (~95° ± 4° to ~40° ± 5°) (*p* < .05) of the contact angle after the interaction with saliva (Figure 3B,C). However, there was no significant difference in the wettability parameters of the investigated membranes when comparing saliva samples collected from the different donors.

Roughness parameters revealed distinct values for the membranes tested due to the different membrane chemical compositions and membrane manufacturing processes. After saliva interaction, roughness parameters, membrane thickness, and membrane density did not show significant alterations, except by the membrane density in biodegradable membranes (Table 1).

**TABLE 1:** Physical characteristics of GBR membranes before and after saliva interaction.

Physical characteristics	Biodegradable membrane	d‐PTFE	e‐PTFE
Roughness parameter (Ra—μm) before saliva interaction	3.5 ± 1.1	6.5 ± 3.35	0.9 ± 0.25
Roughness parameter (Ra—μm) after saliva interaction	3.3 ± 1.5	6.0 ± 3.6	0.9 ± 0.1
Roughness parameter (Rq—μm) before saliva interaction	3.8 ± 1.5	7.1 ± 3.0	1.1 ± 0.10
Roughness parameter (Rq—μm) after saliva interaction	3.2 ± 1.2	6.75 ± 3.1	1.0 ± 0.50
Membrane thickness before saliva interaction (mm)	0.25 ± 0.02	0.23 ± 0.02	0.25 ± 0.01
Membrane thickness after saliva interaction (mm)	0.24 ± 0.02	0.23 ± 0.02	0.25 ± 0.01
Membrane density before saliva interaction (g/cm^3^)	0.4 ± 0.02	1.7 ± 0.01	1.3 ± 0.01
Membrane density after saliva interaction (g/cm^3^)	0.75 ± 0.02*	1.7 ± 0.01	1.3 ± 0.01

*Note*: Obs: * symbolizes statistical significance compared to the same groups of membranes before and after saliva interaction (*p* < .05).

### 3.2 Total salivary protein quantification, total DNA extraction, and 16S rRNA extraction

**FIGURE 4:**
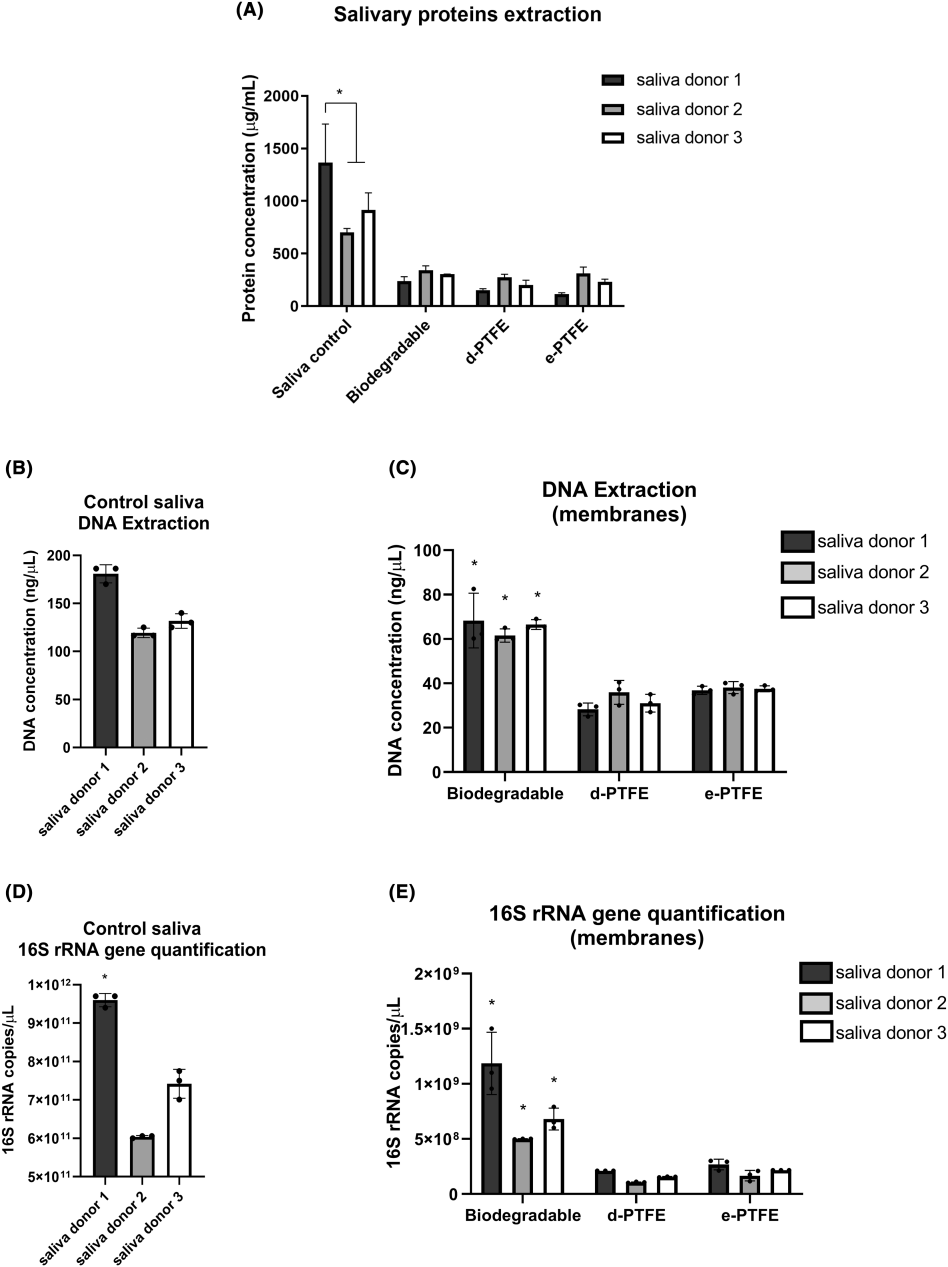
Total quantification of saliva proteins (A). Total DNA extraction and total 16S rRNA gene quantification from saliva control samples without inclusion of membranes (B and D). Total DNA extraction (C) in different membrane groups following the detachment protocol. Quantification of the total bacterial load (16S rRNA gene) adhered/absorbed to different membrane groups following the detachment protocol (E). * symbolizes statistical significance compared with other groups (*p* < .05).

The investigation of total salivary proteins, total DNA, and total 16S rRNA on saliva samples without the inclusion of membranes (controls) showed statistically significant higher values (*p* < .05) for the saliva donor 1 compared with donors 2 and 3 in the total salivary proteins quantification and total 16S rRNA extraction, therefore, evidencing dissimilar saliva composition from the different donors (Figure 4A,B,D). No significant difference was found in total quantification measurements of salivary proteins between biodegradable and non‐biodegradable membranes (Figure 4A). However, total DNA extraction and 16S rRNA gene quantification revealed significantly higher levels for the biodegradable membranes compared with PTFE membranes in the three saliva donors. (Figure 4B–E).

### 3.3 Human gingival fibroblasts proliferation and LDH activity

**FIGURE 5:**
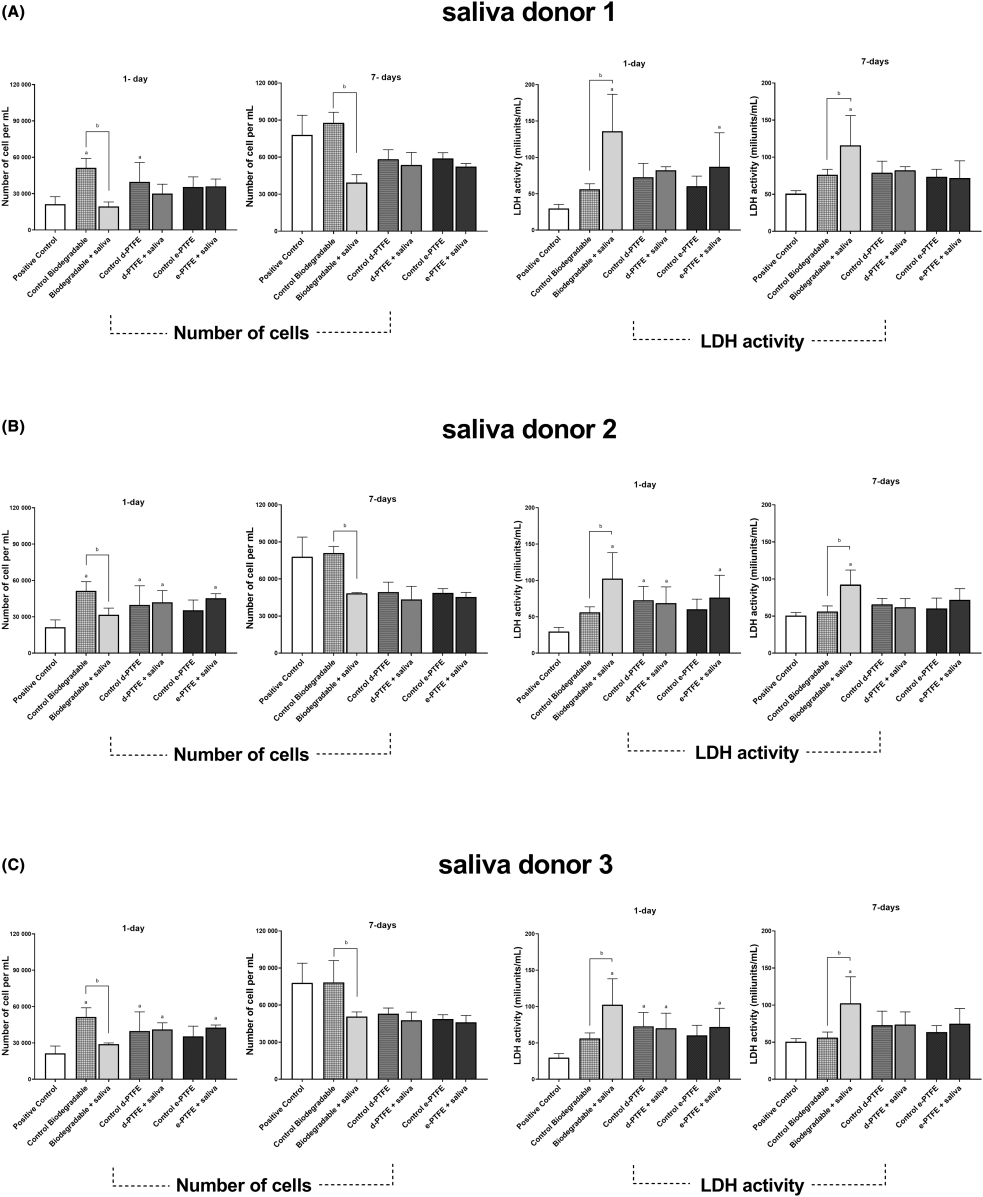
Quantification of HGF‐1 cell numbers (left) and LDH activity (right) in saliva‐contaminated and non‐contaminated membranes after 1 and 7 days of the three different saliva donors (A‐C). Positive control—HGF‐1 culture without membranes. “a” symbolizes statistical significance (*p* < .05) between experimental groups and positive control. “b” symbolizes statistical significance (*p* < .05) between control and saliva‐contaminated samples within the same membrane group.

Biological assays using human gingival fibroblasts (HGF‐1) cultured on the surface of the GBR membranes showed a significant effect of saliva contamination on cell proliferation and viability. Firstly, prior to saliva contamination, all membrane types cultured with HGF‐1 cells showed an increase in the number of cells after 24 h compared to the control—HGF‐1 cell culture without membranes. Cell numbers were significantly higher (*p* < .05) in biodegradable membranes and d‐PTFE membranes prior to saliva‐membrane interaction compared to positive control without membranes for all donors (Figure 5A–C), and in both d‐PTFE and e‐PTFE membranes upon the interaction with saliva samples collected from donors 2 and 3 (Figure 5B,C). In contrast, saliva interaction (for all the saliva donors) with biodegradable membranes negatively influenced cell numbers. Similarly, a significant increase (*p* < .05) in LDH comparing the same groups for the three saliva donors was detected only in the biodegradable membrane group (Figure 5A–C). After 7 days of culture, the results presented a resemblance to 1 day of culture, demonstrating only a statistically significant reduced number of cells and increased LDH activity for contaminated biodegradable membranes compared with non‐contaminated membranes.

### 3.4 MC3T3‐E1 cell line proliferation, LDH activity, and ALP activity

**FIGURE 6:**
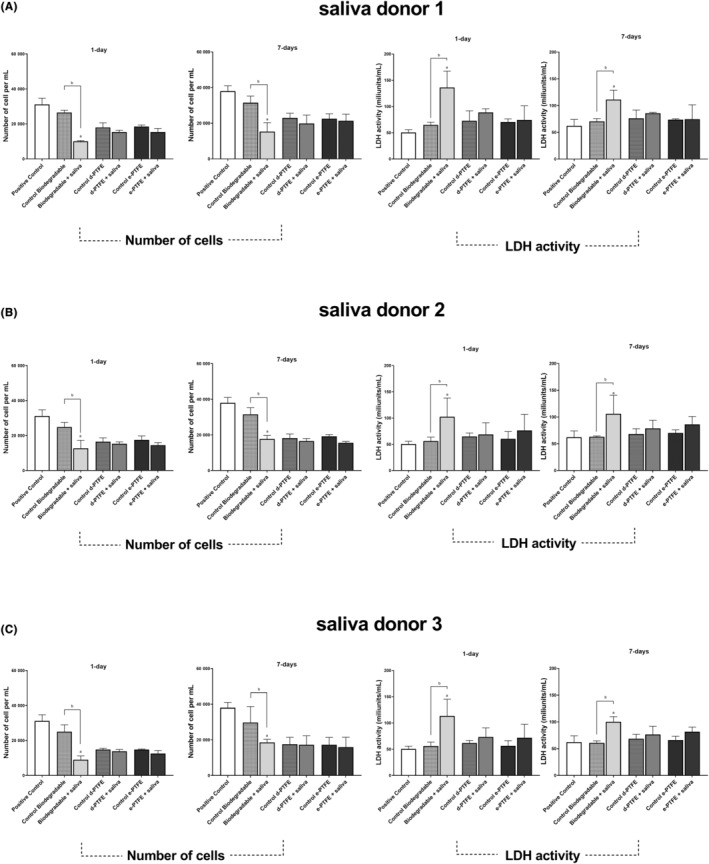
Quantification of MC3T3‐E1 cell numbers (left) and LDH activity (right) in saliva‐contaminated and non‐contaminated membranes after 1 and 7 days of the three different saliva donors (A‐C). Positive control—MC3T3‐E1 culture without membranes. “a” symbolizes statistical significance (*p* < .05) between experimental groups and positive control. “b” symbolizes statistical significance (*p* < .05) between control and saliva‐contaminated samples within the same membrane group.

Differently from the outcomes noticed with gingival fibroblast assays, MC3T3‐E1 cells demonstrated higher cell numbers in control experiments without membranes compared with all cell cultures including membranes for all saliva donors. Biodegradable membranes interacted with saliva revealed to reduce significantly (*p* < .05) the number of MC3T3‐E1 cells and increase significantly (*p* < .05) the LDH activity compared with non‐contaminated biodegradable membranes at 24 and 168 h (Figure 6A–C). Meanwhile, e‐PTFE and d‐PTFE membranes did not show significant differences after saliva interaction for all donors, however, a slight decrease in the number of cells was possible to be noticed.

Relative activity of ALP did not show statistical differences at 24 h of investigation for any saliva donor. However, after 7 days, a significant (*p* < .05) reduction in ALP activity was noticed for saliva‐contaminated biodegradable membranes compared with non‐contaminated biodegradable membranes; these results were consistent for the three saliva donors (File S1).

## 4 DISCUSSION

Membranes for GBR have been applied in several oral procedures involving bone‐related regeneration, periodontal‐related surgeries, and implant placement.^1^, ^8^, ^23^, ^24^ Such clinical surgical situations are challenging due to the presence of saliva excreting from major glands, minor glands, and minimal glands present in the gingival tissues.^12^, ^25^ Saliva interaction with biomaterials in surgical events is unwanted and often occurs in the early stages of the biomaterial insertion before suture closure. To evaluate experimentally the effect of early isolated saliva interaction, a short, 10‐min interaction with human saliva on three different types of membranes used in GBR procedures was studied, simulating an interaction time before the suture closure, necessary for manipulation of membranes during the surgical procedures. However, it is important to point out that GBR membranes applied in clinical procedures face an interaction with oral fluids including blood, cells, and saliva, not necessarily being exposed for 10 min to saliva. Therefore, the results should be evaluated taking into consideration our experimental hypotheses, and the data presented here may not exhibit identical outcomes compared to a fully clinical methodology.

Saliva conditions (collection methods, application methods, or patient habits) and/or altered saliva composition induced by healthy and unhealthy oral conditions, or uncommon metabolic status are significant factors that may alter outcomes in saliva research.^9^, ^13^, ^26^, ^27^, ^28^ Here, we used saliva from three different donors not presenting any kind of unhealthy oral conditions or metabolic dysfunction, targeting to perform our study without strong biases caused by different severities of diseases or pharmacological effects in saliva composition. The application of saliva collected from humans classified with healthy conditions allows the development of a “groundwork” study that can be comparable with other types of saliva in future studies and serve as a baseline for research on this subject. Even though this study prioritized saliva donors with optimistic oral conditions was possible to notice differences in the rates of DNA extraction and bacterial 16S rRNA extraction (controls) of the pure saliva of these donors, evidencing the presence of distinct saliva compositions in humans with similar oral care. Moreover, the application of human saliva provides significantly higher molecular detail and clinically relevant data when compared to studies performed with artificial saliva^29^ generating evidence with higher significance for clinical application. Therefore, findings in this study include pioneering data for saliva research related to biomaterials. Additionally, the protocol that we used for detachment and analysis of saliva content, immediately after 10 min of saliva contamination, offers a pioneer and reproducible step‐by‐step methodology with a high potential for standardization in the saliva research field. As a limitation, this study has a limited number of donors (triplicate) reducing the power of the results for clinical translation. Moreover, saliva composition has an important variability between donors with distinct oral conditions which may cause differences in the outcomes exposed here.^30^ Future clinical research is planned to include multiple donors and/or multicentric collecting sites of saliva allied with donors presenting altered oral health conditions to check similarities to the results presented in this study; and to investigate biological events such as proteomics, spectrum of microbiome adhered to membranes, and gene expression in a complete clinical set generating improved biological accuracy of data.

Membrane's surface properties such as topography, chemical composition, and wettability are designed to promote exceptional response of cells and oral tissues to accelerate the healing process.^31^, ^32^, ^33^ Roughness measurements of GBR membranes demonstrated distinct parameters between the membrane groups due to the different manufacturing processes and materials. However, following saliva contamination the roughness parameters were not significantly altered, suggesting that the salivary layer may be extremely thin or spaced. Non‐biodegradable membranes made of PTFE showed a significant reduction of hydrophobic conditions after saliva contamination in the wettability experiments, meanwhile, biodegradable membranes revealed a slight loss of hydrophilicity. These findings suggest the formation of a thin film of saliva on the membrane surfaces, potentially changing the interaction profile between the fluids and the substrate. SEM images after saliva contamination evidenced the external interaction with saliva showing impurities on the membrane surfaces, indicating surface energy modification. Alterations in wettability properties of biomaterials after saliva contamination have been demonstrated previously^34^, ^35^, ^36^, ^37^ and are aligned with the findings of this study. Therefore, it can be implied that the wettability changes and possible impurity adherence caused by saliva contamination interfere with the surface properties of the membranes and may imply on the biocompatibility response.

Several studies have demonstrated the effects of saliva‐contaminated dental materials on stimulation of bacterial attachment, penetration, and proliferation.^38^, ^39^, ^40^, ^41^ The formation of a salivary pellicle may provide nutrients for bacterial behavior and induce different chemical binds linking bacteria to the substrate.^38^, ^39^, ^40^ An intense interaction between membrane surfaces and saliva components was noticed after saliva exposure to the membrane types in our investigation. Biodegradable membranes showed to rapidly absorb saliva content and had increased amounts of DNA and 16S rRNA gene copy numbers, demonstrating a high susceptibility of these membranes to saliva contamination. This corroborates and adds evidence to the previously introduced concept of the negative effects of saliva contaminating biomaterials and promoting bacterial adhesion. Saliva‐embedded biodegradable membranes could foster a favorable environment for bacterial growth and survival, as shown by the total DNA and 16S rRNA gene copy numbers.

Regarding cellular reactions to the membranes evaluated in this study, a soft tissue cell line (HGF‐1) and a hard tissue cell line (MC3T3‐E1) were chosen for the biological assays due to the specific niche of surgical placement of GBR membranes. GBR procedures are aimed to guide and stimulate bone regeneration allied with defense from the invasion of soft tissue and external cells; therefore, the membranes are allocated exposing one surface side to the hard tissue area and the other surface side to the soft tissue area.^42^ Turri et al.^7^ revealed in a preclinical study the potential of GBR membranes to promote a bioactive function on the bone regeneration underlying the membrane surfaces, differently from a common passive barrier. On the other hand, the contamination of GBR membranes with saliva might affect this relevant property by changing biocompatible functions; and past studies lack this scientific evidence.

Based on the meticulous fundamental evaluation previously described, the biocompatibility of biodegradable saliva‐contaminated membranes was found to reduce significantly. The higher amount of saliva content absorbed/adhered by the biodegradable membranes correlated to a reduced viability of HGF‐1 and MC3T3‐E1 cells and led to an increase of LDH activity, as well as, a reduction in ALP activity after 7 days. Additionally, higher values of LDH were detected before and after saliva contamination for all membrane groups compared to the non‐membrane control in the fibroblast experiment. These findings are supported by the higher number of cells found on membrane surfaces compared with the non‐membrane control. Previous studies testing the viability of both soft and hard tissue cells have corroborated the negative effects of saliva contamination on cell proliferation and behavior reducing the number of cells and modifying positive characteristics.^43^, ^44^, ^45^ The lower number of proliferated fibroblasts on the membranes observed after saliva interaction may suggest an impaired soft tissue regeneration underlying the membrane side exposed to the mucosal tissue in clinical procedures, inducing a fragile flap healing and risks for dehiscences after suture. Interestingly, all the membrane types investigated in this study showed a higher number of HGF‐1 cells when cultured over the non‐saliva‐contaminated membrane surfaces compared with the cell culture control without membranes while the MC3T3‐E1 cells showed the opposite result. This outcome may demonstrate the accelerated profile of gingival cells in proliferating closer to GBR membranes while bone‐related cells need an extended time, which supports the requirement of using GBR membranes in clinical regenerative procedures to maintain the bone tissue protected from soft tissue invasion.^46^, ^47^ Despite the substantial distinct topographies of the three membrane groups, surface morphology and roughness did not show to clearly affect the viability of cells. The chemical composition of biodegradable membranes (collagen) was suggested to be the main factor impacting the higher number of cell proliferation when compared to PTFE membranes.

From a clinical perspective, the experiments performed with saliva collected from individuals presenting excellent oral conditions and no systemic complications evidenced some negative results for the membrane properties after early interaction, suggesting that a future interaction with saliva collected from compromised or systemic‐altered patients might increase these negative outcomes. The findings shown here highlight the importance of avoiding and controlling the early interaction of saliva with biodegradable membranes to prevent possible changes in physicochemical and key biological properties. As‐manufactured membrane properties showed to change characteristics after saliva interaction, which in turn, may lead to negative unplanned clinical outcomes. The interaction of biodegradable membranes with saliva, even for short periods, might generate absorption and adherence of surrounding bacteria exhibited in the oral environment, impacting on possible infection of the surgical site and/or reduced tissue regeneration. On the other hand, it is important to emphasize that the biodegradable membrane used in this study is made of crosslinked collagen, while other membrane types present on the market have a non‐crosslinked design.^48^ The internal structure of collagen membranes may lead to different outcomes regarding saliva contamination and future studies are needed to address this question. Lastly, the clinical assumptions described here should be confirmed by a complete clinical study implementing a large sample of saliva donors.

## 5 CONCLUSIONS

Despite the limitations of this experimental study, a pioneering protocol for the investigation of early saliva interaction with membranes used in GBR procedures was developed. We revealed that biodegradable membranes easily interact with saliva which favors physicochemical alterations; binding of higher amount of total DNA and 16S rRNA gene; reduced survivability of HGF‐1 and MC3T3‐E1 cells; increased LDH activity; and reduction of ALP activity; confirming the negative impact of early saliva interaction with biodegradable membranes, while PTFE membranes were not critically affected. These findings suggest compromised biomaterial properties and might induce reduced capability of tissue regeneration, hence, clinical protocols involving biodegradable membranes in oral‐ and periodontal‐related procedures may need to be refined accordingly. Nevertheless, future in vivo and clinical studies based on the protocol established here are needed to investigate/compare profound molecular evidence and the variability of saliva from different donors presenting distinct systemic conditions.

## FUNDING INFORMATION

This study was supported by the Osteology Research Scholarship, Advanced Research Traineeship 22‐018 (Osteology Foundation, Lucerne, Switzerland), and Hjalmar Svensson Foundation. This research was supported by the Fundação para a Ciência e Tecnologia (UIDB/04279/2020).

## CONFLICT OF INTEREST STATEMENT

The authors report no conflict of interest in this study.

## PATIENT CONSENT STATEMENT

Saliva donors agreed to use their saliva in scientific experiments and for publishing scientific data by signing a consent agreement.

## Supporting information


File S1

## Data Availability

The data that support the findings of this study are available from the corresponding author upon reasonable request.
