# Supplementary material for: Unveiling the consequences of early human saliva contamination on membranes for guided bone regeneration
Source: J Periodontal Res. 2024 Apr 22;59(6):1196–209. doi: 10.1111/jre.13266 (PMC11626699; doi:10.1111/jre.13266)
Supplement: Supplementary file 1 — File S1 [file JRE-59-1196-s001.docx]

**Unveiling the consequences of early human saliva contamination on membranes for guided bone regeneration.**

**Supporting material file S1:**


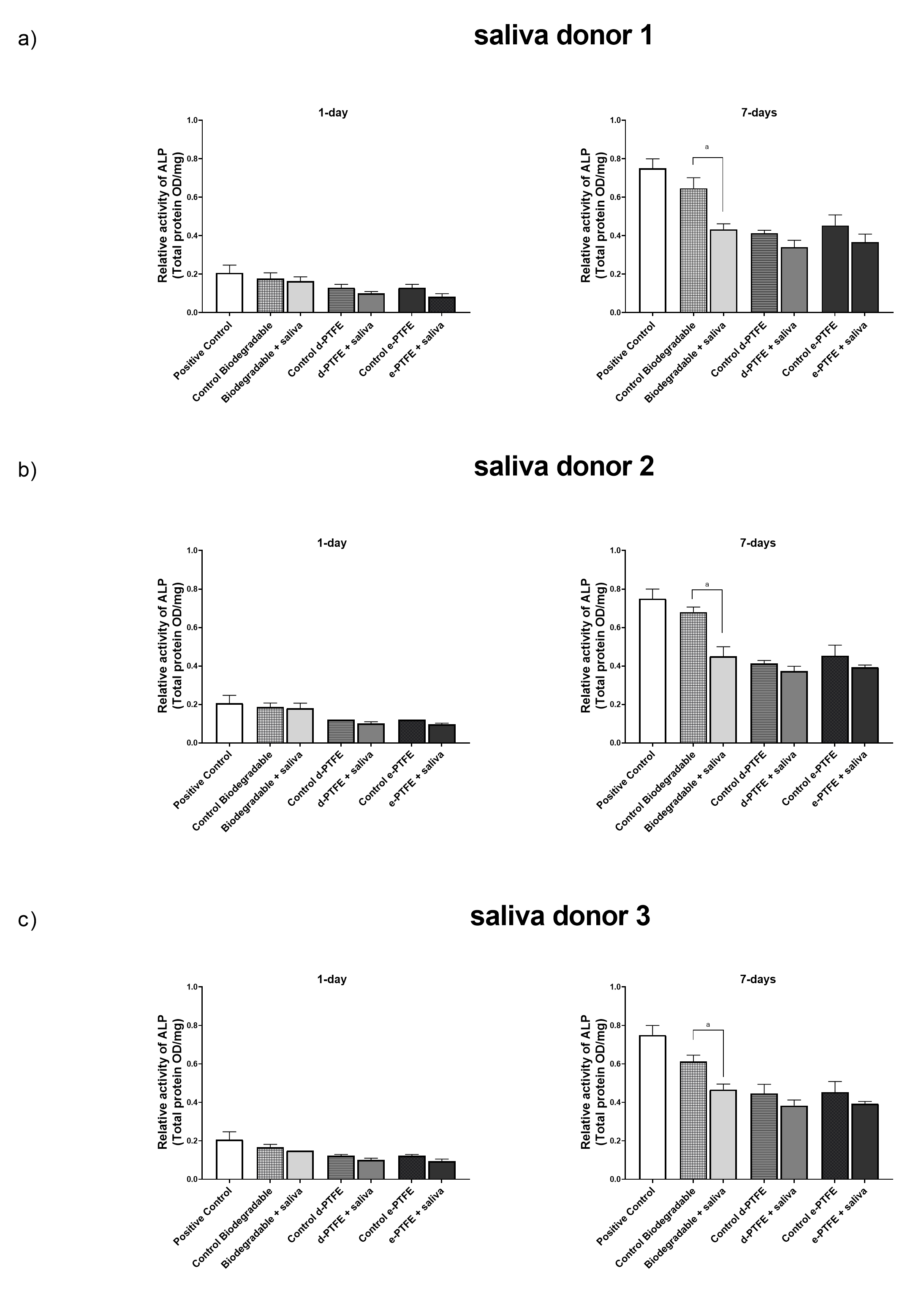
Quantification of ALP activity on MC3T3-E1 cells in saliva-contaminated and non-contaminated membranes after 1-day and 7-days of the three different saliva donors. Positive control – MC3T3-E1 culture without membranes. “a” symbolizes statistical significance (p<0.05) between control and saliva-contaminated samples within the same membrane group.
